# Cap‑assisted intrapapillary cholangioscopic electrohydraulic lithotripsy for impacted papillary stones

**DOI:** 10.1055/a-2971-4154

**Published:** 2026-10-26

**Authors:** Zhiyong Xiong, Bo Liu

**Affiliations:** 1 Department of Hepatobiliary, Pancreatic and Splenic Surgery 144991 Third Affiliated Hospital of Sun Yat-Sen UniversityGuangzhouGuangdongChina

Impacted papillary stones are challenging to manage because conventional extraction techniques may fail. Needle‑knife papillotomy is frequently required to expose and remove the impacted stone; however, the technique is demanding, carries a risk of adverse events, and inevitably disrupts papillary anatomy and sphincter function. 1​ Cholangioscopy‑guided electrohydraulic lithotripsy (EHL) has emerged as an effective treatment for difficult biliary stones, and transparent cap‑assisted cholangioscopy has recently been reported to facilitate stone therapy. 2​

Here, we describe a cap‑created water-filled cholangioscopic platform that enabled intrapapillary EHL and papilla-preserving removal of an impacted papillary stone ( Video 1 ).

**Video 1** Cap‑assisted intrapapillary cholangioscopic electrohydraulic lithotripsy for impacted papillary stones. A transparent cap mounted on the cholangioscope created a localized water-filled chamber within the papillary cavity, enabling intrapapillary electrohydraulic lithotripsy. Initial fragmentation created sufficient space for selective biliary access and subsequent intraductal lithotripsy. Complete stone clearance was achieved under direct visualization without fluoroscopy, while preserving papillary integrity.


A 54‑year‑old woman presented with abdominal pain and obstructive jaundice. Computed tomography demonstrated choledocholithiasis with an impacted stone at the major papilla ( Fig. 1 ). Duodenoscopy confirmed an impacted papillary stone at the papillary orifice ( Fig. 2 ).

**Fig. 1:**
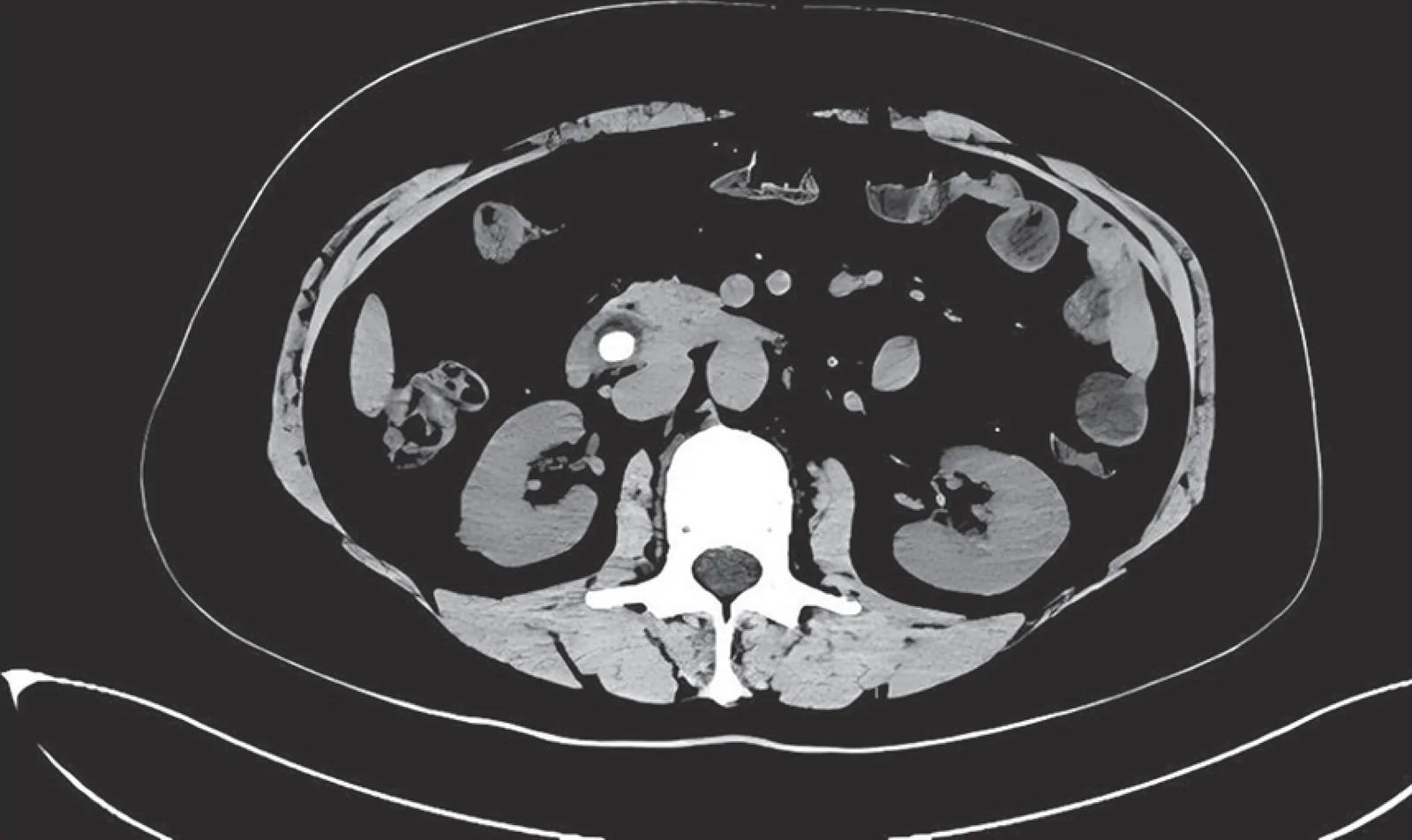
Computed tomography demonstrating an impacted stone located at the major papilla.

**Fig. 2:**
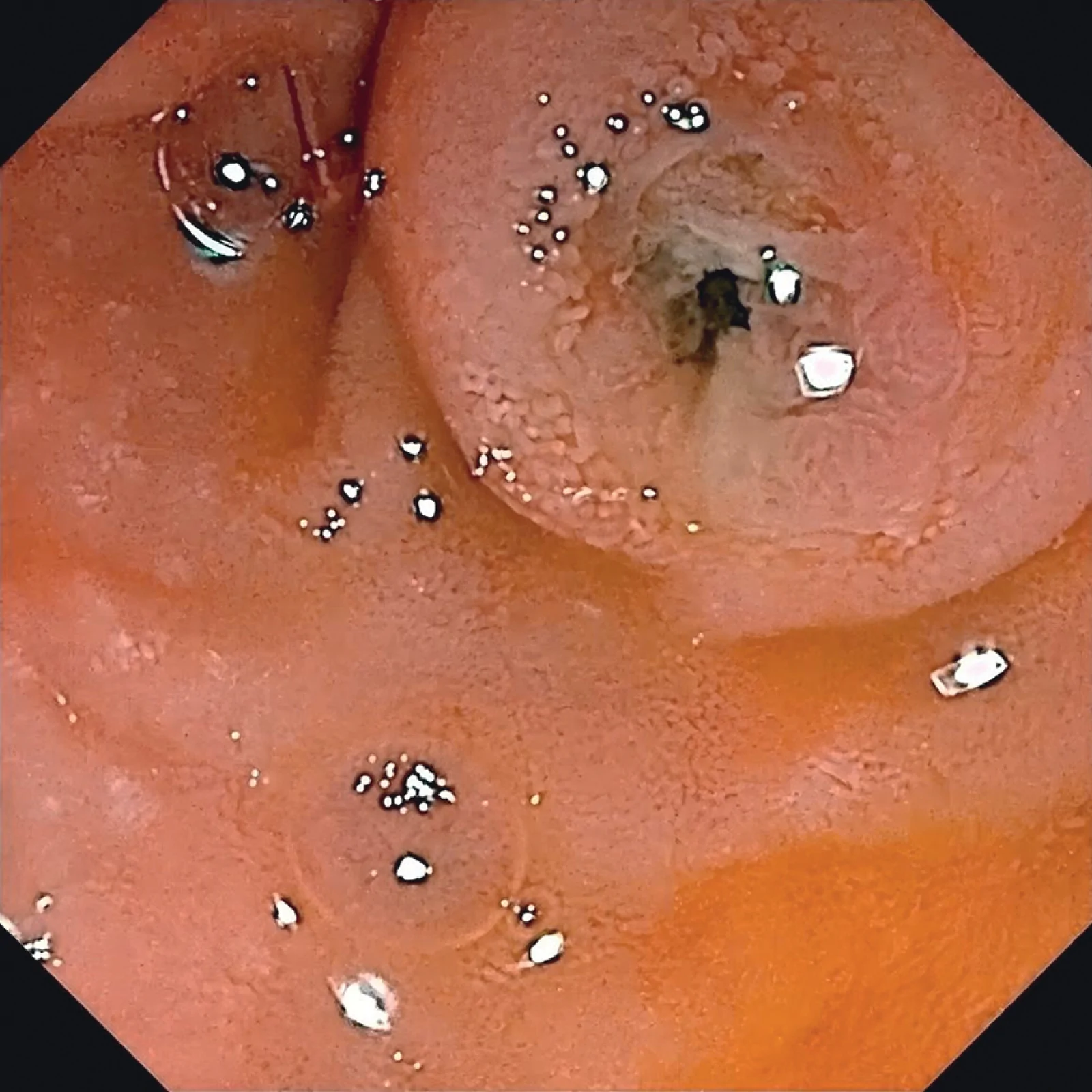
A duodenoscopic view showing a stone impacted at the papillary orifice before intervention.

To facilitate intrapapillary lithotripsy, a transparent cap was mounted on the distal tip of the cholangioscope. Continuous irrigation within the cap created a localized water-filled chamber at the papillary orifice, providing a confined aqueous environment for EHL activation.

Simultaneous duodenoscopic and cholangioscopic visualization enabled the real-time assessment of both external papillary changes and intrapapillary stone fragmentation. Initial EHL was performed at the papillary orifice within the cap-created aqueous chamber ( Fig. 3 ). Partial fragmentation allowed stone debris to exit through the papillary orifice and created sufficient space within the papillary cavity. The cholangioscope was then advanced into the papillary cavity, where direct intrapapillary EHL was performed.

**Fig. 3:**
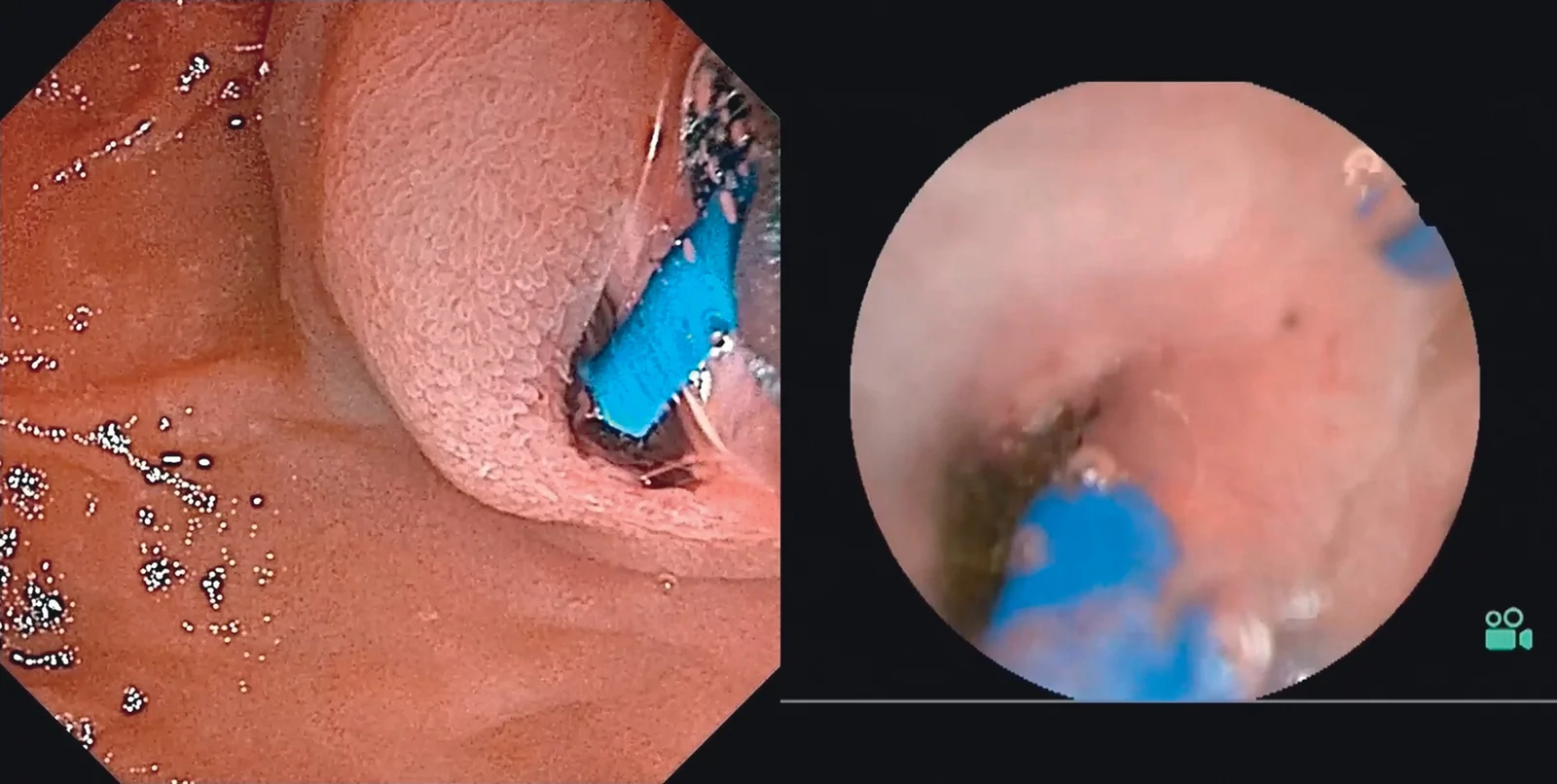
Simultaneous duodenoscopic and cholangioscopic visualization demonstrating the cap-created water-filled chamber and intrapapillary electrohydraulic lithotripsy.

After selective biliary access was achieved, the cholangioscope was advanced into the common bile duct, where additional EHL was performed to treat residual biliary stones. Complete stone clearance was confirmed by cholangioscopic inspection. Final endoscopic evaluation demonstrated preserved papillary anatomy without sphincterotomy ( Fig. 4 ).

**Fig. 4:**
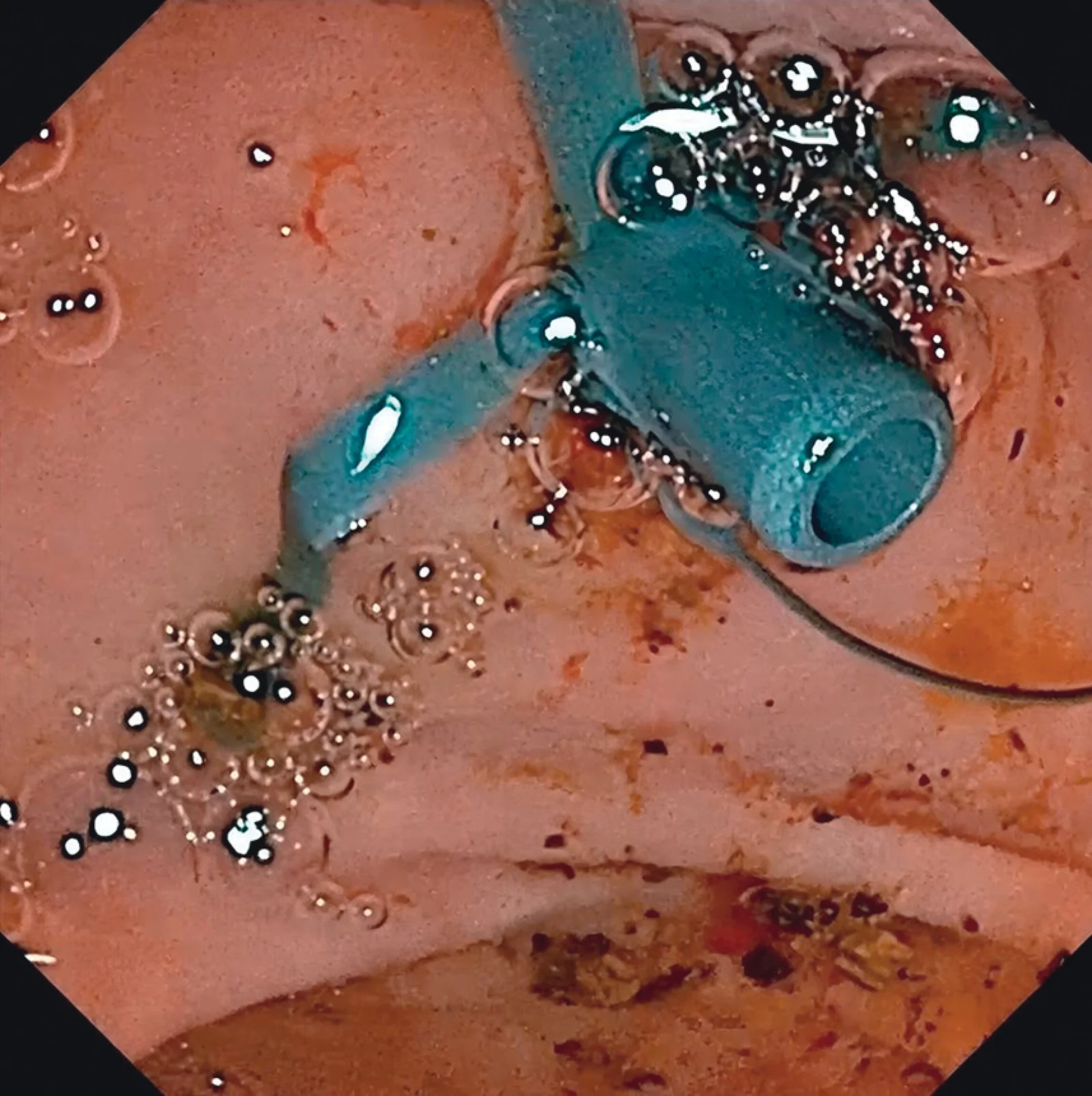
Final endoscopic inspection demonstrating preserved papillary anatomy without sphincterotomy after complete stone clearance.

The entire procedure was completed under radiation-free ERCP without fluoroscopy.

This case demonstrates that a cap-created water-filled cholangioscopic platform can establish a stable aqueous environment for intrapapillary EHL, enabling effective stone fragmentation, selective biliary access, complete stone clearance, and preservation of papillary integrity. This approach represents a novel sphincter-preserving alternative for managing impacted papillary stones.

Endoscopy_UCTN_Code_TTT_1AR_2AH

## Declaration of GenAI Use

ChatGPT (OpenAI) was used solely for language editing and improvement of manuscript readability. All scientific contents, data interpretation, and final manuscript approval were performed by the authors.
